# Supplementary material for: Major Surgical Trauma Impairs the Function of Natural Killer Cells but Does Not Affect Monocyte Cytokine Synthesis
Source: Life (Basel). 2021 Dec 22;12(1):13. doi: 10.3390/life12010013 (PMC8777869; doi:10.3390/life12010013)
Supplement: Supplementary file 1 [file life-12-00013-s001.zip › Müller et al. Supplemental Figures REV.pptx]

## Slide 1
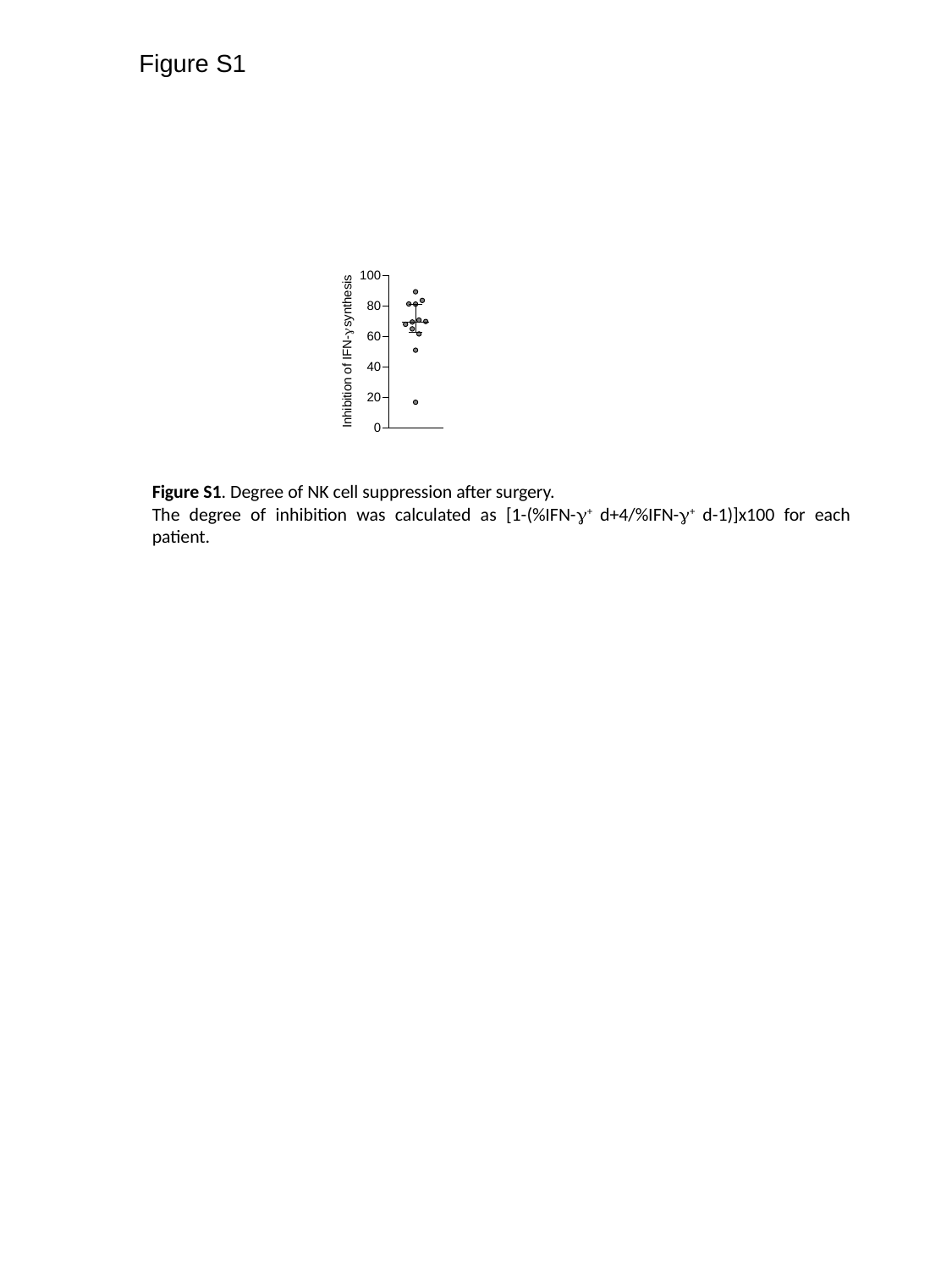

Figure S1
Figure S1. Degree of NK cell suppression after surgery.
The degree of inhibition was calculated as [1-(%IFN-g+ d+4/%IFN-g+ d-1)]x100 for each patient.

## Slide 2
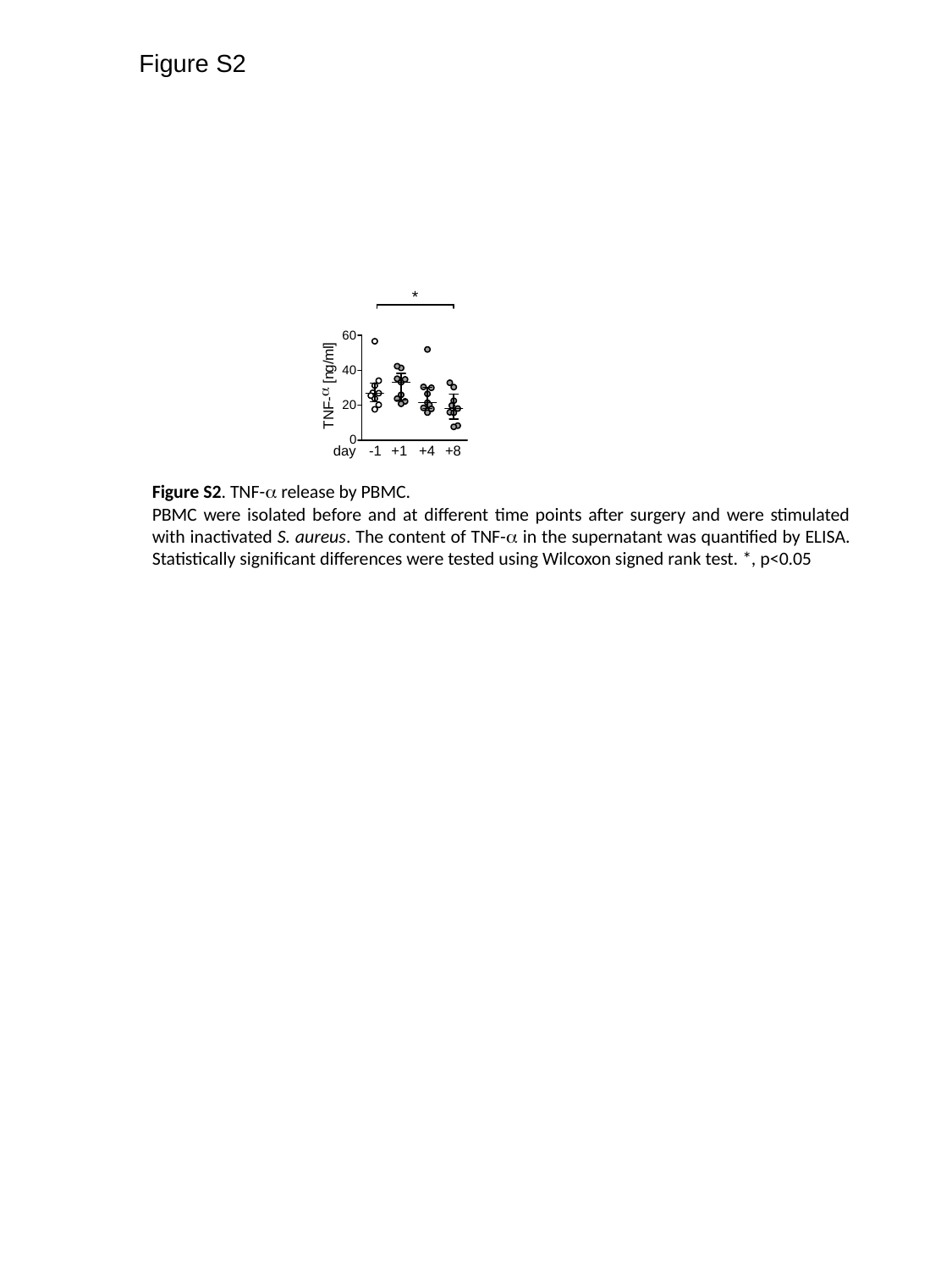

Figure S2
-1
day
+4
+8
+1
Figure S2. TNF-a release by PBMC.
PBMC were isolated before and at different time points after surgery and were stimulated with inactivated S. aureus. The content of TNF-a in the supernatant was quantified by ELISA. Statistically significant differences were tested using Wilcoxon signed rank test. *, p<0.05

## Slide 3
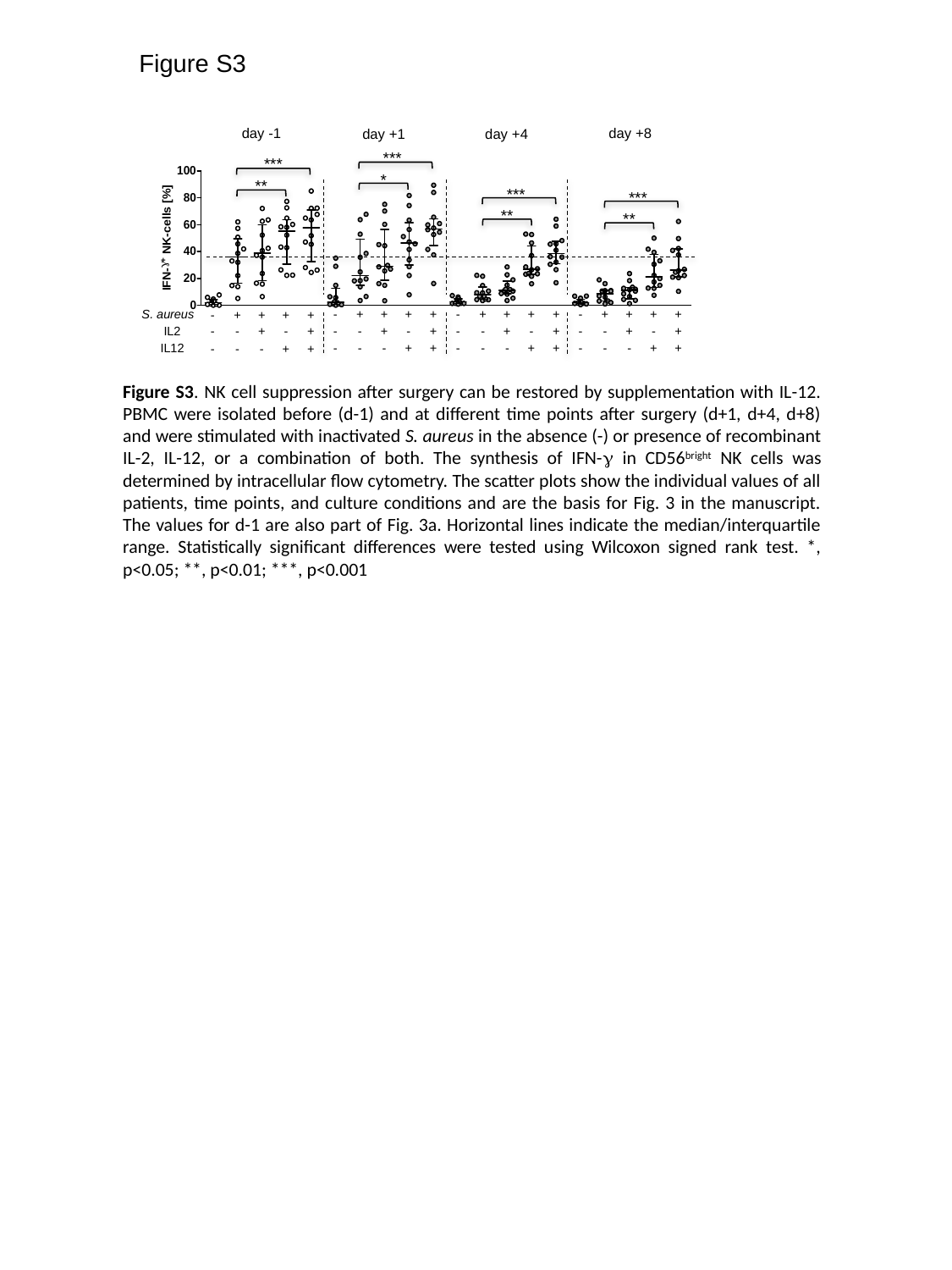

Figure S3
day -1
day +8
day +1
day +4
***
***
*
**
***
***
**
**
-
+
+
+
+
-
+
+
+
+
-
+
+
+
+
S. aureus
-
+
+
+
+
-
-
+
-
+
-
-
+
-
+
-
-
+
-
+
IL2
-
-
+
-
+
-
-
-
+
+
-
-
-
+
+
-
-
-
+
+
IL12
-
-
-
+
+
Figure S3. NK cell suppression after surgery can be restored by supplementation with IL-12. PBMC were isolated before (d-1) and at different time points after surgery (d+1, d+4, d+8) and were stimulated with inactivated S. aureus in the absence (-) or presence of recombinant IL-2, IL-12, or a combination of both. The synthesis of IFN-g in CD56bright NK cells was determined by intracellular flow cytometry. The scatter plots show the individual values of all patients, time points, and culture conditions and are the basis for Fig. 3 in the manuscript. The values for d-1 are also part of Fig. 3a. Horizontal lines indicate the median/interquartile range. Statistically significant differences were tested using Wilcoxon signed rank test. *, p<0.05; **, p<0.01; ***, p<0.001

## Slide 4
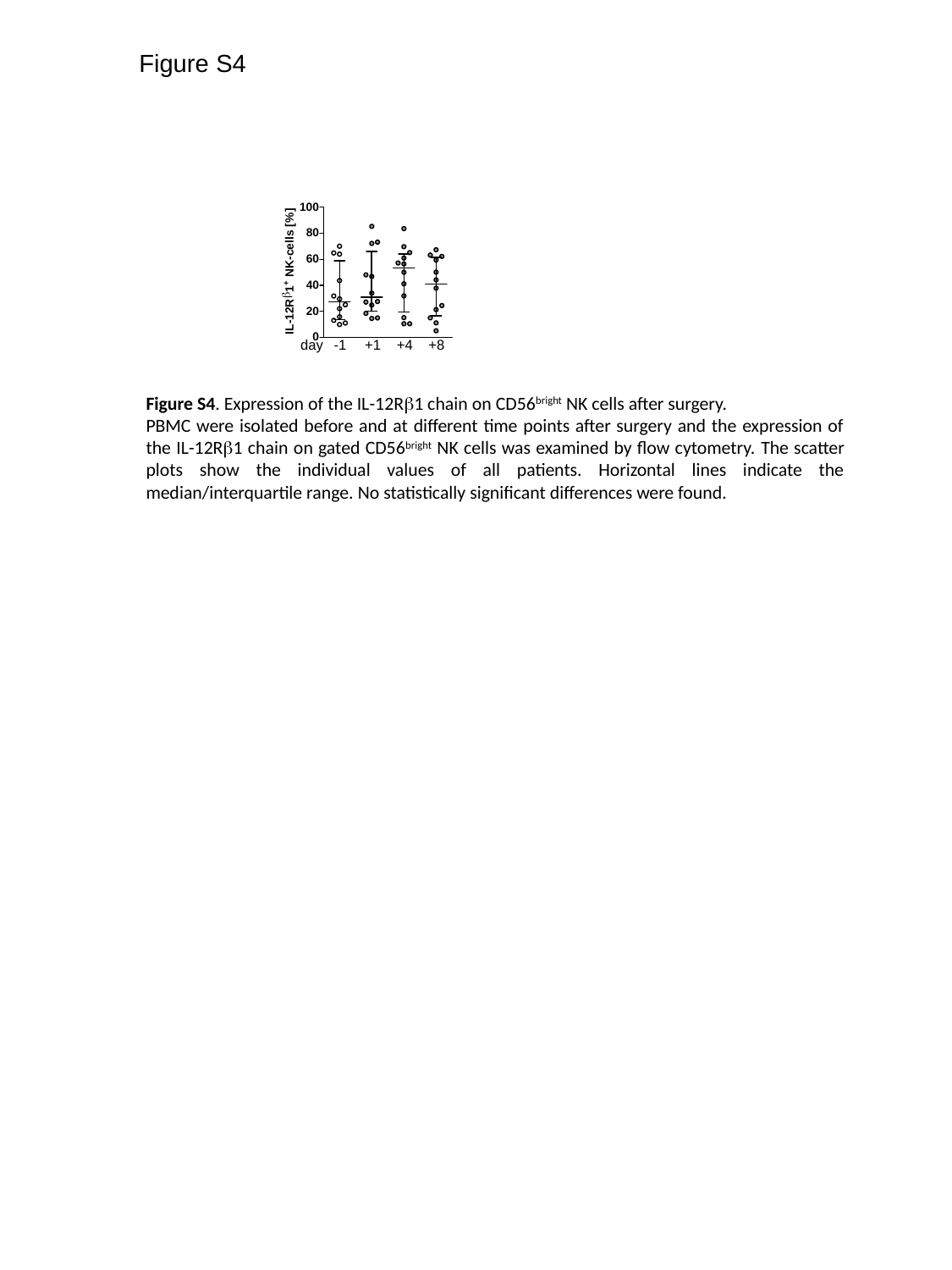

Figure S4
day
-1
+1
+4
+8
Figure S4. Expression of the IL-12Rb1 chain on CD56bright NK cells after surgery.
PBMC were isolated before and at different time points after surgery and the expression of the IL-12Rb1 chain on gated CD56bright NK cells was examined by flow cytometry. The scatter plots show the individual values of all patients. Horizontal lines indicate the median/interquartile range. No statistically significant differences were found.

## Slide 5
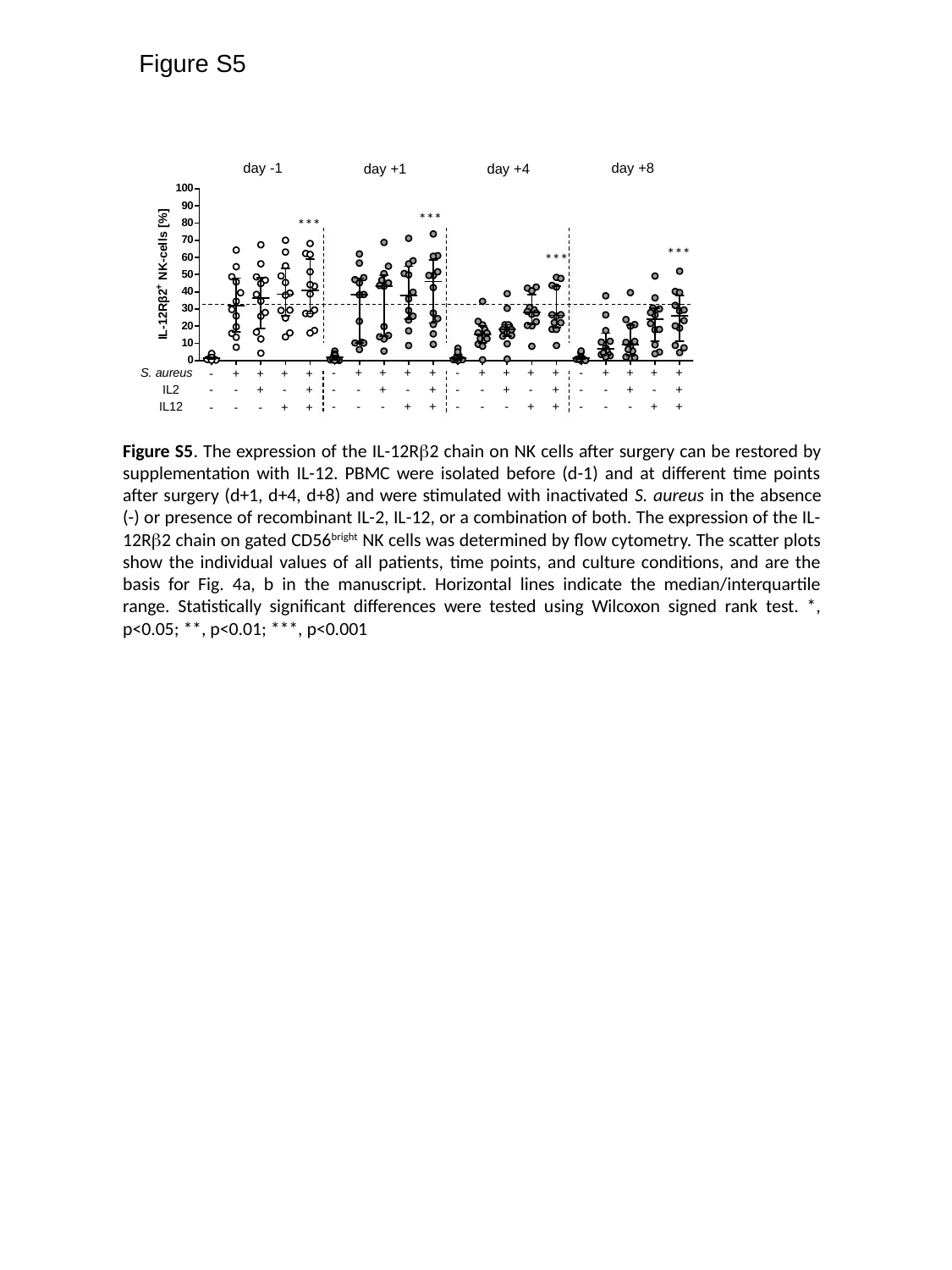

Figure S5
day -1
day +8
day +1
day +4
***
***
***
***
-
+
+
+
+
-
+
+
+
+
-
+
+
+
+
S. aureus
-
+
+
+
+
-
-
+
-
+
-
-
+
-
+
-
-
+
-
+
IL2
-
-
+
-
+
-
-
-
+
+
-
-
-
+
+
-
-
-
+
+
IL12
-
-
-
+
+
Figure S5. The expression of the IL-12Rb2 chain on NK cells after surgery can be restored by supplementation with IL-12. PBMC were isolated before (d-1) and at different time points after surgery (d+1, d+4, d+8) and were stimulated with inactivated S. aureus in the absence (-) or presence of recombinant IL-2, IL-12, or a combination of both. The expression of the IL-12Rb2 chain on gated CD56bright NK cells was determined by flow cytometry. The scatter plots show the individual values of all patients, time points, and culture conditions, and are the basis for Fig. 4a, b in the manuscript. Horizontal lines indicate the median/interquartile range. Statistically significant differences were tested using Wilcoxon signed rank test. *, p<0.05; **, p<0.01; ***, p<0.001
