# Supplementary material for: Major Surgical Trauma Impairs the Function of Natural Killer Cells but Does Not Affect Monocyte Cytokine Synthesis
Source: Life (Basel). 2021 Dec 22;12(1):13. doi: 10.3390/life12010013 (PMC8777869; doi:10.3390/life12010013)
Supplement: Supplementary file 1 [file life-12-00013-s001.zip › Table S1.pdf]

Table S1. Correlation of the concentration of GDF-15 1 d after surgery with IL-6, CRP, myoglobin, duration of surgery, and blood transfusion. GDF-15 levels in the serum obtained 1 d after surgery was tested for significant correlation using spearman correlation.

Correlation of GDF-15 on d+1 with	**Spearman r**	**p-value**
IL-6 d+1	0.5804	0.0521
CRP d+1	0.3280	0.3221
CRP d+2	0.2857	0.5008
Myo d+1	-0.098	0.7664
Duration of surgery	0.3020	0.2521
Volume of blood transfusion	0.4256	0.4256
